# Expressing Status and Correlation of ARID1A and Histone H2B on Breast Cancer

**DOI:** 10.1155/2016/7593787

**Published:** 2016-01-21

**Authors:** Yan Wu, Yan Gu, Shanyu Guo, Qiancheng Dai, Wei Zhang

**Affiliations:** ^1^Department of Surgery, The Ninth People's Hospital, School of Medicine, Shanghai Jiao Tong University, Shanghai 200011, China; ^2^Department of Surgery, Zhoupu Hospital of Pudong District, Shanghai, China

## Abstract

ARID1A is one of the important cancer-related genes and regulates transcription of certain genes by altering chromatin structure. Inactivated mutations and decreased expression of ARID1A gene have been reported in several kinds of cancer. Histone H2B is a major component of chromatin and encoded by HIST1H2BE. The goal of the study is to evaluate expressing status of ARID1A and H2B as well as their correlation on breast cancer. Gene expression profiles of ARID1A and H2B on Oncomine database are analyzed. Tissue microarray of breast cancer was used for examination of ARID1A and H2B expression by immunohistochemistry. As a result, the disagreement of ARID1A expression was found, while HIST1H2BE expression is elevated in 4 out of 5 datasets on Oncomine database. There were 15 cases (20%) of breast cancers that were positive for ARID1A. Fifty-eight out of 75 cases of breast cancer (77.3%) were highly expressed for H2B protein and 17 cases (22.7%) were low expressed for H2B protein. All cases with ARID1A expression are overlapped with H2B high expression. Among 15 cases with ARID1A and H2B coexpression, 13 are invasive ductal carcinoma and 2 are mucinous carcinoma. Our results indicate that ARID1A gene may be involved in carcinogenesis of some subtypes of breast cancer.

## 1. Introduction

ARID1A (AT-rich interactive domain 1A) is one of the important cancer-related genes by large-scale cancer genome sequencing in recent years. Mutations in the chromatin remodeling gene ARID1A have recently been identified in the majority types of cancer, such as gastric cancer, colon cancer, bladder cancer, ovarian cancer, liver cancer, and breast cancer [1–6]. In stomach cancer, ARID1A mutations result in lost expression and obtain a better prognosis [7, 8]. However, Huang and coworkers found that mutations of ARID1A gene in primary liver cancer cause an enhanced invasiveness and metastatic ability [3]. Inactivated mutations and hypermethylation of promoter of ARID1A gene have been reported in breast cancer by several reports [9]. However, the precise mechanisms of ARID1A gene in carcinogenesis are largely unknown.

ARID1A is located on chromosome 1p35.3, a region frequently deleted in human cancers, which encodes a member of the SWI/SNF family, whose members have helicase and ATPase activities and are thought to regulate transcription of certain genes by altering the chromatin structure [10, 11]. ARID1A has a DNA-binding domain that can specifically bind an AT-rich DNA sequence known to be recognized by a SNF/SWI complex at the beta-globin locus. The C-terminus of the protein can stimulate glucocorticoid receptor-dependent transcriptional activation. Recently, Li and colleagues found that histone H2B is a functional target of ARIDIA, which is involved in histone modifications [12].

H2B is a basic nuclear protein that is responsible for the nucleosome structure of the chromosomal fiber in eukaryotes. One report disclosed that H2B monoubiquitylation is a 5′-enriched active transcription mark [13]. Slowinski and coworkers noticed that there was a high correlation between H2B mRNA level and nuclear division index as well as histone labeling index in human glioma cell lines. Histone H2B mRNA level and histone labeling index may be a useful molecular predictor of the tumor response to radiation treatment in gliomas of the same histological grade [14]. Moreover, Hao and coworkers revealed that high level of serum histone H2B could predict a poorer prognosis of gastric cancer [15]. To clarify the possible relation of ARID1A and H2B, we analyzed the expressing status of ARID1A and H2B on breast cancer.

Oncomine is a web-based microarray database. It currently contains 674 datasets including 73327 samples from different types of tumors and provides free access to all researchers. Oncomine integrates high-throughput cancer profiling data across a large volume of cancer types, subtypes, and experiments so that target expression can be assessed online [16–18]. In present study, we screened the expression status of ARID1A and HIST1H2BE (H2B encoding gene) by Oncomine database and then examined the protein expression of ARID1A and histone H2B simultaneously on tissue microarray of breast cancer.

## 2. Materials and Methods

### 2.1. Oncomine Database Analysis

To get the outline of the ARID1A and H2B expression pattern, we searched the ARID1A and H2B mRNA levels in human cancers using datasets from the publicly available Oncomine database (http://www.oncomine.org).

### 2.2. Tissue Microarray and Reagents

Tissue microarray (TMA) of breast cancer (OD-CT-RpBre01-003) was purchased from OUTDO Biotech Co., LTD. (Shanghai, China), which contains 80 cases of breast cancer who are aged from 33 to 81 years (average 55 years). Ten of them have paired adjacent normal breast tissue. Among them, 55 cases are invasive ductal carcinoma, 6 invasive lobular carcinoma, 9 mucinous carcinoma, 4 medullary carcinoma, 3 lipid-rich carcinoma, and 3 ductal carcinoma in situ.

### 2.3. Ethics Statement

Written informed consent in the study was obtained from all participants. The study protocol was approved by the ethics committee of OUTDO Biotech Co., LTD., Shanghai.

### 2.4. Immunohistochemistry for ARID1A and H2B

TMA was dewaxed and hydrated using alcohol. Antigen was retrieved by citrate buffer, and endogenous hydrogen peroxide was blocked with 3% hydrogen peroxide solution. Mouse antihuman ARID1A monoclonal antibody (1 : 50, SC-32761, Santa Cruz, USA) and rabbit antihuman H2B monoclonal antibody (1 : 1200, ab52599, Abcam, USA) were dropped and incubated at 37°C for 1 h, respectively, and then washed 3 times with 1x PBS for 5 min. Then, EnVision two-step reagents (Dako) were incubated at 37°C for 30 min. DAB was used for coloration, and hematoxylin was used for nuclear staining with positive signals observed as yellow or brown patches. Both ARID1A and H2B are expressed in the nucleus or nucleus/cytoplasm was judged as positive staining. In order to guarantee the reliability of the experiment, we use a previous confirmed gastric cancer as positive control and remove primary antibody as a negative control. TMA was scored according to the percentage of cells stained positively, and the intensity of the staining. The percentage of stained cells was scored on a scale of 0 (<10%), 1 (10%–30%), 2 (31%–50%), and 3 (51%–100%). Stain intensity was scored on a scale of 0 (negative), 1 (weak), 2 (moderate), and 3 (strong). A cumulative staining score ranging from 0 to 9 was used for statistical analysis and was obtained by multiplying intensity scores with cell percentage scores. Total staining score was graded as weakly positive (<4 scores) and strongly positive (over 6 scores).

### 2.5. Statistical Analysis

Statistical analysis was performed by using the program package SPSS 15.0. The measurement data were analyzed by* t*-test, and numeration data were analyzed using the *χ* ^2^ test or Fisher's exact test. Differences were considered significant at *P* < 0.05.

## 3. Results

### 3.1. mRNA Expressing Pattern of ARID1A and HIST1H2BE on Oncomine Database

**Figure 1:**
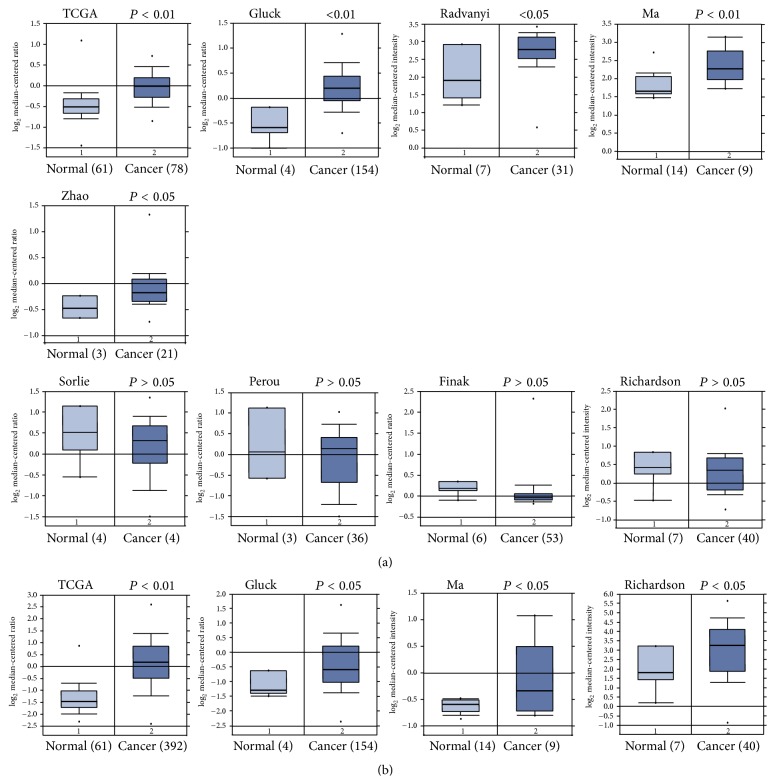
Search for ARID1A and HIST1H2BE mRNA expression of human breast cancer in Oncomine database. (a) Analysis of ARID1A mRNA levels in human breast cancer tissues on different datasets. (b) The mRNA levels of HST1H2BE of breast cancer groups are elevated in different datasets.

In 13 datasets for ARID1A, the mRNA expression of ARID1A was differed between several datasets. ARID1A mRNA was found significantly elevated in human breast cancer tissues compared with normal tissues in datasets from TCGA, Radvanyi, Ma, Gluck, and Zhao's groups, while ARID1A mRNA was decreased in human breast cancer tissues compared with normal tissues in Sorlie, Perou, Finak, and Richardson's datasets (Figure 1(a)). However, we noticed that the cases examined in every group are very different. For instance, in Gluck's group, there are only 4 normal controls against 154 cancer cases [19–22]. There is obvious disagreement between different datasets. We searched 5 datasets for HIST1H2BE expression. The expression of HIST1H2BE was elevated in cancer compared to that in normal tissue on 4 out of five datasets (Figure 1(b)). One remaining dataset (Turashvili dataset) revealed no difference of HIT1H2BE expression between breast cancer group and normal tissue group [19, 21, 23].

### 3.2. The Protein Expression of ARID1A and H2B on Tissue Microarray

**Figure 2:**
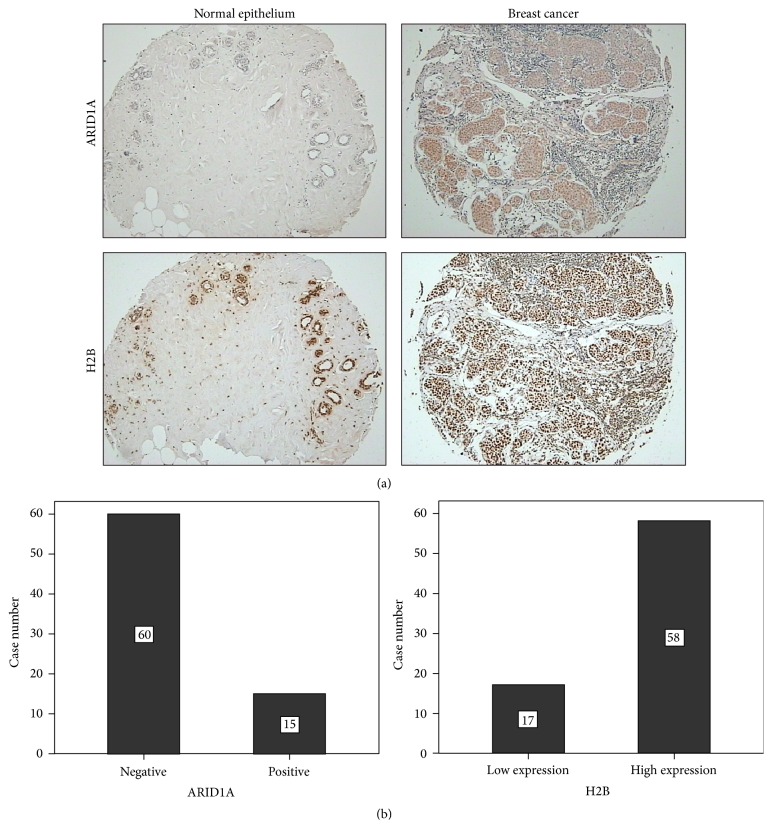
Immunohistochemistry of ARID1A and H2B in breast cancer tissues and corresponding normal tissues. (a) Top: ARID1A was not expressed in normal mammary epithelium and expressed in breast cancer tissue. Down: H2B was expressed in both normal mammary epithelium and breast cancer tissue (100x). (b) Bar chart of immunohistochemical staining results for ARID1A and H2B.

**Figure 3:**
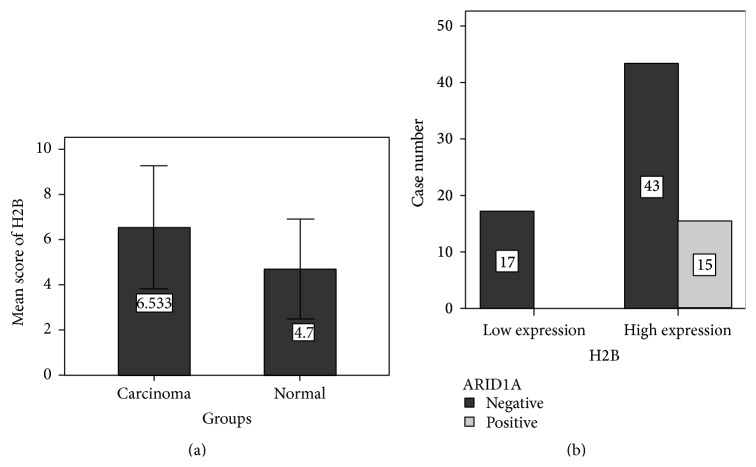
Immunohistochemical scoring and correlation analysis of H2B and ARID1A. (a) The mean score of H2B protein in breast cancer tissues was significantly higher than that in normal mammary epithelium (*P* < 0.05). (b) The protein expressions of ARID1A and H2B are closely related (*P* = 0.019). All 15 cases with ARID1A expression were overlapped with the highly expressed H2B.

A total of 75 cases are included in the final analysis except for five shedding points from tissue arrays. The age of patients was from 33 to 81 years (average 55 years). Ten of them have paired adjacent normal breast tissue. In this group, 51 cases of tumor were primary breast cancer without metastasis and 24 cases with metastasis in axillary lymph nodes. We found weak or lost ARID1A expression in normal epithelial cells of breast. There were 60 (80%) cases of breast cancers with negative expression of ARID1A. Only 15 cases (20%) of breast cancers were positively expressed for ARID1A. H2B protein was expressed on normal mammary epithelium (with scores from 4 to 6). In breast cancer, 58 cases (77.3%) were highly expressed for H2B protein (with scores from 6 to 9) and 17 cases (22.7%) were low expressed for H2B protein (Figure 2). The mean score of H2B protein in breast cancer tissues was significantly higher than that in normal mammary epithelium (*P* < 0.05, Figure 3(a)).

**Figure 4:**
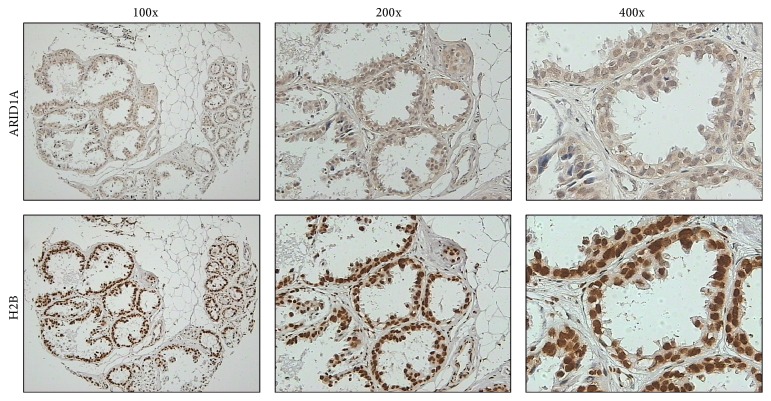
The coexpressing observation of ARID1A and H2B protein in one cancer case. Top: ARID1A protein expression in serial section. Down: H2B protein expression in serial sections. Left panel: low power magnification (100x); middle panel: moderate power magnification (200x); right panel: high power magnification (400x).

**Figure 5:**
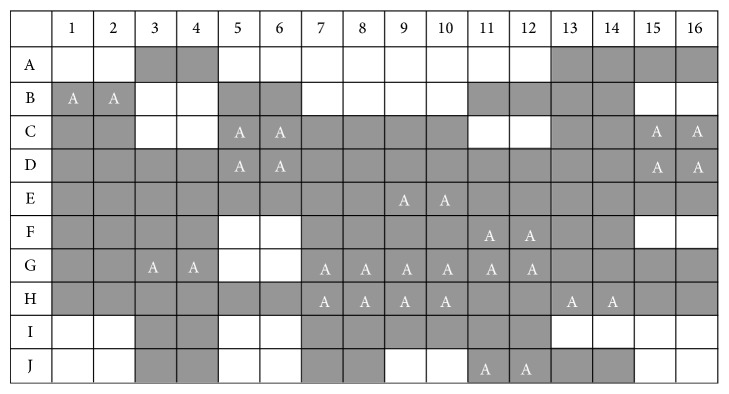
The schematic diagram of breast cancer tissue microarray used in current study. Each breast cancer case has two tissue points on the same line. The gray grid in the microarray represented strong positivity for H2B protein. The capital letter A in the grids represented positivity for ARID1A protein. All 15 cases with ARID1A expression were overlapped with the highly expressed H2B.

We compared multiple clinicopathological parameters with ARID1A and H2B expression. There is no significant difference between ARID1A or H2B expression with patients' age, location, histological types, and axillary lymph node status. However, we noticed that the protein expressions of ARID1A and H2B are closely related to each other (*P* = 0.019, Table 1). In nonparametric correlation analysis, the expression of ARID1A and H2B is positively related to Spearman's *R* 0.28 (*P* = 0.009). It suggested that the expression of ARID1A is often companied by the high expression of histone H2B in a subset of breast cancers (Figure 3(b)).  Figure 4 presented expression of ARID1A and H2B on the same case of invasive ductal carcinoma in serial sections of tissue array. The positive correlation of protein expression of ARID1A and H2B was well presented (Table 2). Both ARID1A and H2B presented nucleus or nucleus/cytoplasm expressing pattern. The expression status of ARID1A and H2B in serial sections of tissue arrays was summarized in Figure 5, in which each case has two tissue points on the same line. The gray grid in the array represented strong positivity for H2B protein, while the white capitalized letter A represented positivity for ARID1A protein. All 15 cases with ARID1A expression were overlapped with the highly expressed H2B. Although there was no statistic significance between ARID1A and H2B expression with histological types, we noticed that the 13 cases with ARID1A expression belong to invasive ductal carcinoma and the remaining 2 cases are mucinous carcinoma.

## 4. Discussion

Decreased expression of ARID1A on breast cancer has been found in several studies. As to reasons, it may be caused by the genomic mutations, whereas the promoter methylation may also be involved in the decline of gene expression [2, 9, 24]. Here, we examined ARID1A protein expression in 75 cases of breast cancer and corresponding normal epithelium. We noticed that the ARID1A protein expression in normal breast epithelium is weak or lost. The expression of ARID1A was found in 20% cases of breast cancer. The histology of ARID1A positive cases is invasive ductal carcinoma and mucinous carcinoma. By Oncomine database analysis, the expression of ARID1A gene is inconsistent. In TCGA dataset, elevated ARID1A gene expression was found in invasive carcinoma and mucinous carcinoma. TCGA group's finding is consistent with our result. It suggested that ARID1A gene may involve in carcinogenesis in some subtypes of breast cancer.

ARID1A protein is a DNA-binding protein, mainly involved in the regulation of chromatin remodeling in cell nucleus [25]. ARID1A is a member of the ARID family of DNA-binding proteins and a subunit of human SWI/SNF-related complexes. Histone H2B is a main component of chromatin. Li and colleagues reported that histone H2B is a direct target of ARID1A. ARID1A gene is involved in activation and expression of histone H2B through ubiquitin modification [12]. In current study, we firstly analyzed the expressing status and relationship of ARID1A and histone H2B in serial sections of tissue microarray. Because the protein expression of ARID1A was limited in 20% cases of breast cancer, we think that ARID1A may be not master gene in carcinogenesis of breast cancer. It may participate in development of some subtype of breast cancer.

Histone H2B is moderately expressed in normal breast epithelium and highly expressed in 70% cases of breast cancer. However, the precise role of H2B protein in breast carcinogenesis is largely unknown. We carefully analyzed the correlation between ARID1A protein expression and H2B protein expression and found that all of ARID1A expressing cases are accompanied by high expression of H2B protein. The coexpression of ARID1A and H2B suggested that ARID1A may be involved in the histone modification in some subtypes of breast cancer. However, the exact role of H2B in breast cancer is not clear yet. Our data revealed that H2B high expression is frequent in breast cancers, and we need to investigate the role of H2B in breast cancer further.

## Figures and Tables

**Table 1:** Correlation of ARID1A or H2B expression with clinicopathological parameters.

Parameters	ARID1A positive	ARID1A negative	*P* value
Age			
<50	6	24	1.00
>51	9	36	
Histology			
Invasive tubular cancer	10	41	0.118
Invasive lobular cancer	3	3	
Others	2	16	
Axillary node			
Positive	7	17	0.173
Negative	8	43	
Location			
Left side	8	32	1.00
Right side	7	28	
H2B expression			
High	15	43	0.019
Low	0	17	

Parameters	H2B high	H2B low	*P* value

Age			
<50	25	5	0.311
>51	33	12	
Histology			
Invasive tubular cancer	42	9	0.169
Invasive lobular cancer	3	3	
Others	13	5	
Axillary node			
Positive	18	6	0.741
Negative	40	11	
Location			
Left side	31	9	0.971
Right side	27	8	
ARID1A expression			
Negative	43	17	0.019
Positive	15	0	

**Table 2:** The details of clinical pathological information of breast cancer cases used in the study.

Case number	Age	Location	Cancer types^*∗*^	Lymph node metastasis	ER expression	PR expression	ARID1A score	H2B score
1	74	Right	IDC	No	Yes	Yes	0	2
2	50	Left	IDC	Yes	No	Yes	0	6
3	53	Left	IDC	No	Yes	Yes	0	3
4	41	Right	IDC	Yes	Yes	Yes	0	0
5	63	Left	IDC	No	Yes	Yes	0	3
6	48	Right	IDC	Yes	Yes	Yes	0	0
7	48	Right	IDC	No	No	Yes	0	6
8	37	Right	IDC	No	Yes	Yes	0	6
9	74	Right	IDC	Yes	Yes	Yes	6	6
10	44	Right	IDC	Yes	No	No	/	/
11	57	Right	IDC	Yes	No	Yes	0	9
12	54	Left	IDC	No	Yes	Yes	/	/
13	66	Left	IDC	No	Yes	Yes	/	/
14	37	Right	IDC	No	Yes	No	0	9
15	48	Right	IDC	No	Yes	Yes	0	6
16	43	Left	IDC	No	Yes	Yes	/	/
17	42	Left	IDC	Yes	Yes	Yes	0	6
18	52	Left	IDC	Yes	No	Yes	0	6
19	48	Left	IDC	Yes	Yes	Yes	3	6
20	53	Left	IDC	No	Yes	Yes	0	9
21	66	Left	IDC	Yes	Yes	Yes	0	9
22	46	Right	IDC	Yes	Yes	Yes	0	3
23	63	Left	IDC	No	Yes	Yes	0	9
24	51	Left	IDC	No	No	No	0	9
25	53	Left	IDC	No	Yes	Yes	0	6
26	71	Left	IDC	No	No	Yes	0	9
27	37	Right	IDC	Yes	Yes	Yes	4	6
28	77	Right	IDC	Yes	No	No	0	6
29	59	Left	IDC	No	No	Yes	0	9
30	58	Left	IDC	Yes	Yes	Yes	0	9
31	43	Right	IDC	No	No	No	0	6
32	57	Left	IDC	No	Yes	Yes	3	9
33	39	Right	IDC	No	Yes	Yes	0	6
34	57	Left	IDC	No	No	No	0	9
35	70	Right	IDC	No	Yes	Yes	0	6
36	47	Left	IDC	No	No	No	0	9
37	52	Left	IDC	Yes	Yes	Yes	6	9
38	53	Right	IDC	Yes	Yes	Yes	0	9
39	45	Left	IDC	Yes	No	No	0	9
40	42	Left	IDC	No	Yes	Yes	0	9
41	71	Left	IDC	No	No	Yes	0	9
42	44	Right	IDC	No	No	No	0	6
43	53	Right	IDC	No	No	No	0	4
44	80	Right	IDC	Yes	Yes	No	0	9
45	44	Left	IDC	No	No	No	3	9
46	70	Left	IDC	No	No	No	3	9
47	77	Right	IDC	No	No	No	0	9
48	51	Left	IDC	No	No	No	0	4
49	51	Left	ILC	No	Yes	Yes	0	4
50	46	Left	ILC	No	Yes	Yes	6	9
51	62	Right	ILC	No	Yes	No	0	4
52	39	Right	ILC	No	Yes	Yes	6	9
53	38	Left	ILC	No	Yes	Yes	3	9
54	59	Left	IDC	Yes	Yes	Yes	3	9
55	45	Left	MUC	No	No	Yes	0	9
56	63	Left	MUC	No	Yes	Yes	0	6
57	49	Right	MUC	No	No	No	0	6
58	48	Left	IDC	No	No	No	0	6
59	50	Right	IDC	No	No	Yes	0	9
60	57	Right	IDC	Yes	Yes	Yes	4	6
61	81	Right	MUC	No	Yes	No	3	9
62	80	Left	MUC	No	Yes	Yes	0	6
63	60	Right	MUC	No	Yes	No	4	6
64	65	Right	MUC	No	Yes	Yes	0	6
65	66	Right	MUC	No	Yes	Yes	0	0
66	65	Right	MUC	No	Yes	Yes	0	9
67	50	Left	MEC	No	No	No	0	0
68	44	Left	MEC	No	No	No	0	6
69	74	Right	MEC	No	Yes	Yes	0	9
70	33	Left	MEC	No	No	Yes	0	9
71	51	Left	LRC	Yes	No	No	0	3
72	55	Left	LRC	No	No	No	0	2
73	56	Right	LRC	Yes	No	No	0	2
74	70	Left	DCI	No	Yes	Yes	0	9
75	65	Right	DCI	No	Yes	Yes	/	/
76	45	Right	DCI	No	Yes	Yes	0	9
77	41	Left	ILC	Yes	Yes	Yes	0	3
78	79	Right	IDC	Yes	Yes	Yes	3	9
79	74	Right	IDC	Yes	No	Yes	0	6
80	51	Left	IDC	No	No	No	0	3

^*∗*^IDC: invasive ductal carcinoma; ILC: invasive lobular carcinoma; MUC: mucinous carcinoma; MEC: medullary carcinoma; LRC: lipid-rich carcinoma; DCI: ductal carcinoma in situ.
